# Health Profile of Construction Workers in Hong Kong

**DOI:** 10.3390/ijerph13121232

**Published:** 2016-12-13

**Authors:** Wen Yi, Albert Chan

**Affiliations:** Department of Building and Real Estate, The Hong Kong Polytechnic University, Hung Hom, Kowloon, Hong Kong, China; albert.chan@polyu.edu.hk

**Keywords:** Hong Kong, construction industry, musculoskeletal pain, clinical examination, questionnaire survey

## Abstract

Construction is a manual, heavy, and complex sector concerning the most fatal accidents and high incidence of occupational illnesses and injuries resulting in days away from work. In Hong Kong, “Pilot Medical Examination Scheme for Construction Workers” was launched in 2014 to detect the health problems of their construction workforce. All registered workers under the Construction Workers Registration Board are eligible to join the scheme. The purpose of this paper is to assess the physical condition, physiological status, and musculoskeletal disorders of 942 construction workers in Hong Kong. This study adopted a two-phase design, which includes a basic medical examination to measure the workers’ physiological parameters, such as blood pressure, resting heart rate, glucose, cholesterol, uric acid, liver function test, and renal function test; as well as a face-to-face interview following the medical examination to collect their demographic information and pain experience. Individual characteristics, including gender, age, obesity, alcohol drinking habit, and sleeping habit influenced the health condition of construction workers. Among the participants, 36.1% and 6.5% of them were overweight and obese, respectively. In addition, 43.0%, 38.4%, 16.2%, and 13.9% of the participants exceeded the thresholds of cholesterol, blood pressure, urea nitrogen, and uric urea, correspondingly. Moreover, 41.0% of the participants suffered musculoskeletal pain, where the most frequent painful parts occur in the lower back, shoulder, knees, leg, and neck. Through these findings, a series of important issues that need to be addressed is pointed out in terms of maintaining the physical well-being and reducing musculoskeletal disorders of construction workers. The finding may have implications for formulating proper intervention strategies for the sustainable development of Hong Kong’s construction industry.

## 1. Introduction

Construction safety is of great concern around the world. Although a great amount of effort has been made to improve safety and health [1,2,3,4], construction remains as one of the most hazardous sectors owing to its association with a high incidence of fatalities and injuries. Global estimates by the International Labor Organization (ILO) reported that more than 60,000 fatalities annually have occurred in the construction sector [5]. The U.S. Bureau of Labor Statistics announced that the highest count of fatal injuries among all sectors during the period of 2014 happened in construction sites, with at least 899 fatal work injuries [6]. Meanwhile, construction workers in the United Kingdom had 217 fatal injuries between 2010 and 2014 [7]. In Hong Kong, the number of accidents in the construction industry stood at 3467 and the total number of fatalities was 20 in 2014 [8]. In Spain, construction is the most dangerous industry, with approximately 350 fatalities per year [9]. These statistics reveal that construction has indeed a high rate of accidents, which result in deaths and financial losses. 

Apart from facing the front-line dangers on a jobsite, construction workers are also subjected to a wide range of chemical and physical hazards (e.g., chemicals, dusts, fibers, noise, radiation, and temperature extremes) throughout the building process [10]. These hazards could lead to acute and chronic illnesses, diseases, cancers, and/or disorders. Chemical hazards come in all forms including dusts, mists, vapors, and gases, which enter the body by inhalation, ingestion, and absorption. These hazards sometimes attack the body at the point of entry, but many pass into the bloodstream or alimentary canal and concentrate their attack on other organs, specifically the lungs, liver, kidney, blood, nervous system, and heart. Physical examination is the assessment of a body to determine overall health status. Several studies on physical examination have been conducted among different occupations and industries. Kawai et al. (1995) conducted a health examination to 816 white collar workers in Japan by testing body mass index (BMI), total cholesterol, uric acid, and glucose [11]. To prevent the cardiovascular diseases of Korean air crews, Choi and Kim (2013) examined the BMI and cholesterol of 326 workers [12]. Haldiya et al. (2005) investigated the BMI, diastolic blood pressure (DBP), and systolic blood pressure (SBP) of 247 salt workers performing tasks close to salt milling plants [13]. Moreover, proof-printing workers who are usually exposed to chemicals are susceptible to cholangiocarcinoma. Thus, Kumagai et al. (2014) evaluated blood parameters of proof-printing workers, including aspartate aminotransferase (AST), alanine aminotransferase (ALT), cholesterol, and glucose metabolism [14]. However, little has been done towards the construction workers for occupational health and safety. The proposed study attempts to fill this gap in assessing the workers’ health status based on a set of physiological parameters measurable by clinical and scientific methods. 

Construction workers have to undertake physically demanding tasks with repetitive motions, awkward postures, high force, and segmental body vibration. Musculoskeletal disorder (MSD) is the most serious cause of occupational injuries in the construction industry. Globally, the symptoms of MSD appeared in approximately 77% of the construction workers, as a result of the cumulative effect of their age, daily operating hours, and work experience [15]. Leung assessed the MSD symptoms of 76.2% of the construction workers in Taiwan who experienced MSD. This result indicated that the most prevalent MSD symptoms manifested involve the shoulder, neck, and low back [16]. Furthermore, a survey on MSD symptoms of US construction workers revealed that at least 77% of the workers experienced at least one MSD symptom, where low back pain was reported as the most frequently experienced symptom [17]. A study in Thailand showed that 57.7% of female construction workers suffered MSD with low back and shoulders as the most common body parts affected [18]. Moreover, MSD is the largest cause of disability, sick absenteeism, and days of work lost, which lead to productivity loss, increased costs on workers’ compensation, insurance, and healthcare. In Sweden, 1342 cases of sick leaves were caused by ergonomic risk factors among construction workers in 2004, taking up approximately 84.8% of all cases of sick leaves. In Canada, the Alberta Construction Safety Association reported musculoskeletal injuries took up 46.8% of the total injury claims and 41.9% of lost time claims [19]. Although interest in assessing the MSD of construction workers has increased, little evidence is available on examining the MSD of Hong Kong workers.

Similar to many countries or regions, Hong Kong’s construction industry is facing several challenges. The construction workforce in Hong Kong is aging. It is reported that 44% of the construction employees were aged over 50 in March 2013 [20]. Furthermore, labor shortage is daunting. In 2012, only 13% of construction workers were under the age of 30, and construction companies had ongoing difficulties in recruiting and retaining young construction personnel [21]. Additionally, construction costs are soaring owing to the aging workforce and labor shortage. Hong Kong was one of the most expensive construction markets in the world because of a number of factors, namely, the shortage of skilled construction employees, rising commodity prices, and increased market demand [21].

The Hong Kong government expressed concerns on this issue and took a series of actions to address the labor problems in the construction industry. Among these approaches, one of the most prominent is the “Pilot Medical Examination Scheme for Construction Workers”(PMES) launched by the Hong Kong Construction Industry Council (CIC) in early March 2014. The purposes of the scheme include early detection of health problems and extraction of useful information on the health conditions of employees in the local construction industry. CIC targeted to examine 10,000 registered construction workers in order to acquire a general health profile of construction workers. This aim of this study is to examine the construction workers’ health profile in terms of BMI, blood pressure, glucose, cholesterol, uric acid, liver function, renal function, and assessed their musculoskeletal pain in terms of pain experience, pain treatment, and its impacts on daily life. During the period of March 2014 and May 2015, a total of 942 construction workers participated in the PMES. This paper presents findings of the initial assessment of 942 workers.

## 2. Participants and Methods

### 2.1. Participants

The participants consisted of registered workers in the construction industry of Hong Kong. The study consists of basic medical examinations and face-to-face interview. Medical examinations were provided by a professional clinic to assess a number of health parameters, including blood pressure, resting heart rate, glucose, cholesterol, uric acid, liver function test, and renal function test. Face-to-face interviews were carried out to measure their demographic information, and pain experience. The PMES was conducted at the construction sites during lunch breaks. The physical test and interview take approximately 15 min per worker. Participants were informed of the purpose and the procedure of the study. Written consent was obtained prior to the study. Confidentiality and anonymity will be assured. Their participation is on a voluntary basis. Data collected in the study will be password-protected and kept centrally in a stand-alone server and will be used for this study only. Only authorized research personnel will have access to the data and the raw data will be destroyed after reporting. The study was approved by the Human Subjects Ethics Application Review System (HSEARS) of authors’ employing institution (Reference number: HSEARS20131218001). All participant information is subject to the current conditions of the Data Protection Act 1998. 

In Hong Kong, a construction worker must be registered before he/she carries out construction works on construction sites. The total number of registered construction workers in Hong Kong is approximately 413,613 [22]. To ensure sufficient statistical power in making inferences about the population, the Penn State’s equation for determining final sample size was used as Equation (1) [23]. A total of 942 construction workers are representative for data analysis. 

Based on:

(1) $$n=\frac{P(1-P)}{\frac{A^{2}}{Z^{2}}+\frac{P(1-P)}{N}}$$

where *n* is the sample size required; *N* is the number of participants in the population; *P* is the estimated variance in population, as a decimal (0.5 for 50–50); *A* is the precision desired, expressed as a decimal (i.e., 0.03, 0.05, and 0.1 for 3%, 5%, and 10%); and *Z* is the based on confidence level: 1.6649 for 90% confidence, 1.96 for 95%, and 2.5758 for 99%. 

Therefore:

(2) $$n=\frac{0.5\times 0.5}{\frac{0.05^{2}}{2.5758^{2}}+\frac{0.5\times 0.5}{413,613}}=656$$

### 2.2. Individual Characteristics and Work-Related Factors

Data on age, sleeping hours, ethnicity, and years of working in construction industry were collected in the face-to-face interviews. Body weight and height of the participants were measured. The body mass index (BMI) was calculated as the ratio of body weight in kilograms and the square of height in meters. The BMI is used to quantify the amount of tissue mass and categorize person as underweight (BMI < 18.5 kg/m^2^), normal (BMI 18.5–22.9 kg/m^2^), slightly overweight (BMI 23.0–24.9 kg/m^2^), moderately overweight (BMI 25.0–29.9 kg/m^2^), or obese (BMI > 30 kg/m^2^) [24]. Smoking habits were measured as “none”, “no more than 35 cigarettes per week”, and “more than 35 cigarettes per week”. Alcohol drinking habits were measured according to the alcoholic treatment criteria adopted by Tung Wah Hospitals [25]. The criteria state that: (i) for men, the appropriate amount of alcohol intake is fewer than three servings per days and fewer than 15 servings per week; and (ii) for women, the appropriate amount is fewer than two servings per days and fewer than 10 servings per week. The participants were further asked whether they do any warm up exercises and whether they do any relaxation exercises before or after doing the construction work every day. 

### 2.3. Clinical Examination

The SBP, DBP, and resting heart rate (RHR) of the participants were measured. The average of two readings was the value used in the analyses. Venous blood was collected from an arm vein following standard sterile techniques. Venous blood samples were collected to obtain serum for laboratory analysis of cholesterol, glucose, uric acid, urea, aspartate transaminase (AST), and alanine transaminase (ALT). All blood samples were transported to the pathology laboratory for analysis. 

### 2.4. Musculoskeletal Pain Assessment

**Figure 1:**
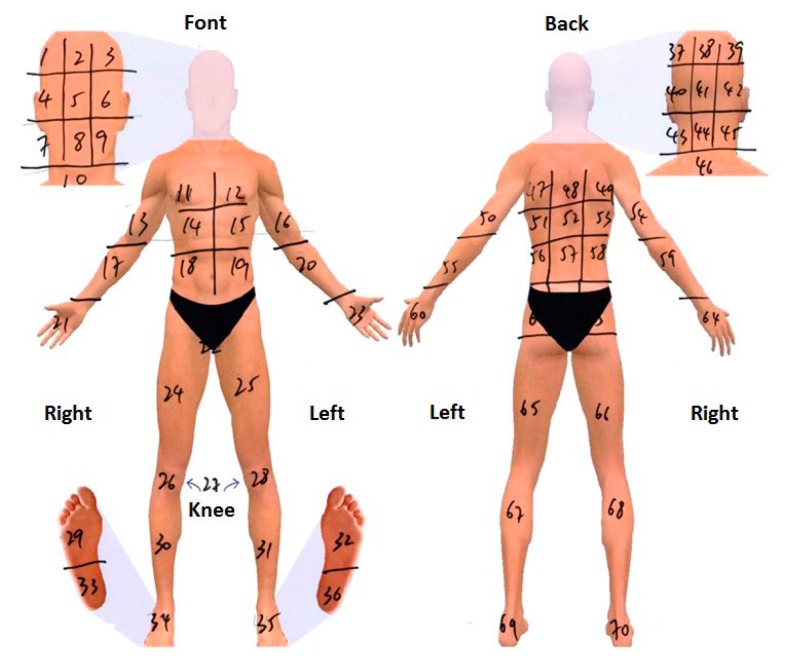
Body regions for the pain location measurement.

To evaluate the musculoskeletal pain, the Pain Experience Questionnaire (PEQ) was adopted to measure workers’ perceived pain symptom. The PEQ, a 9-item, subjective assessment that measures the subjects’ perceived pain, has been shown to be a simple and valid method for pain assessment [26,27]. The participants were asked if they had experienced any of the following types of discomfort or pain that was caused by work or made worse by work in the last three months, including muscular, ligament, tendon, and joint discomfort or pain. Responses were categorized as yes or no (item 1). Moreover, respondents were asked to indicate at which area the discomfort/pain was located (Figure 1). In terms of the location of pain, responses were then sorted into the following categories: head/neck/shoulder, chest/upper back, waist/low back, knee/leg/foot, hand, arm, and whole body (item 2). The participants rated the severity of pain symptoms from a scale of 1 (very mild pain) to 10 (unbearable pain) for the pain they felt over the past 24 h, as well as the pain they felt at that moment (items 3–6). Respondents were also asked if they had ever received any treatment for discomfort/pain. Responses for these items were categorized as follows: ignore, pain killers, cream, acupoint massage, health product, physical therapy, acupuncture, Chinese medicine, exercise, massage or others (item 7). They were also asked for the pain relief after treatment in a scale from 0 (no remission) to 10 (complete remission) (item 8). Furthermore, the participants were asked to rate whether their pain symptoms have affected their mood, walking ability, work ability, relationships, sleeping, and hobbies, on a scale from 1 (strongly disagree) to 10 (strongly agree) (item 9).

### 2.5. Statistical Analysis

Descriptive statistics, such as means and percentages on the main variables were utilized. T-test was conducted to detect whether gender (i.e., male, female) and sleeping habit (i.e., <7 h daily, ≥7 h daily) would significantly affect the health condition (i.e., clinical parameters, musculoskeletal pain) of construction workers. To compare the means of the workers’ health condition in different levels of age (<35 years, 35–50 years, ≥50 years), BMI (underweight, normal, overweight, obese), smoking habit (non-smoker, occasional smoker, daily smoker), and alcohol drinking habit (non-alcohol drinker, occasional alcohol drinker, problematic alcohol drinker), a one-way analysis of variance (ANOVA) was performed to determine if there are any statistically significant differences among the means of groups, followed by Duncan’s multiple range test (DMRT) to differentiate the significance of treatment means. Participants who have medical results exceeding the safety threshold were identified for further analyses. Multiple linear regression was employed to explore the impacts of age, BMI, ethnicity, smoking habit, alcohol drinking habits, and working experience on the musculoskeletal pain. The backward approach was adopted for the initial selection of relevant variables. We used the rating of average pain as an indicator to evaluate the severity of musculoskeletal pain. All of the following statistical analyses were performed with a significance level of 95% (*p* < 0.05) using software program SPSS 21.0 (IBM Corporation, Armonk, NY, USA).

## 3. Results

### 3.1. Demographic Details

**Figure 2:**
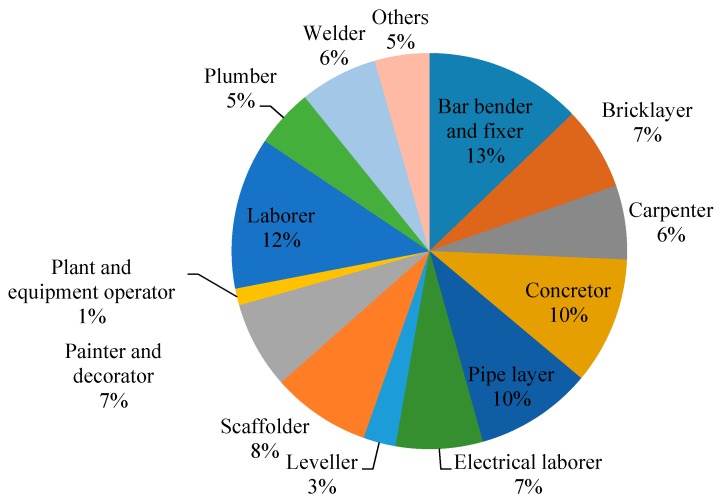
Trade distribution of participants.

Table 1 shows the demographic details of the participants. The majority of the participants were male (86.8%). The mean age of the workers was 45.1 years, ranging from 17 years to 70 years. Most of the workers were between the ages of 22 years to 66 years, whereas those below 22 or above 66 years were scarce. It was found on average that the female workers (50.5 years) were older than the male workers (44.3 years). The mean height, weight, and BMI of the participants were 168.5 cm, 68.5 kg, and 24.3, respectively. It was also found that 2.8% of the workers were underweight (BMI < 18.5 kg/m^2^), 54.6% (BMI of 18.5 kg/m^2^ to24.9 kg/m^2^), 36.1% were moderately overweight (BMI of 25.0 kg/m^2^ to 29.9 kg/m^2^), and 6.5% were obese (BMI > 30 kg/m^2^). Nearly half of the participants were non-smoker (50.9%) and occasional alcohol drinker (52.8%). The mean sleeping hours of participants were 6.9 h. In addition, 89.4% of the construction workers resided permanently in Hong Kong, 9.3% were from Mainland China, and the rest were from South Asia. The mean working experience in the construction industry was 12.9 years, ranging from 0.2 years to 53 years. Trade distributions were illustrated in Figure 2. Participants with a wide spectrum of age, working experience, and trade contributed in this study, thus ensuring a representative sample of frontline workers.

### 3.2. Health Condition 

Table 2 shows the health parameters of the 927 Hong Kong construction workers, in terms of blood pressure, RHR, blood glucose, liver function, urea, cholesterol, and uric acid. 

#### 3.2.1. Blood Pressure and RHR

Following the definition in Poulter et al. (2015), a participant is considered to have hypertension if his/her blood pressure is greater than or equal to 140 mmHg SBP or greater than or equal to 90 mmHg DBP [28]. It was found that 38.4% (356) of the participants’ blood pressure exceed the threshold. The mean resting heart rate of the workers was 76.7 bpm, ranging from 53 bpm to 115 bpm. According to the American Heart Association, the average RHR for adults is 60–100 bpm, and for well-trained athletes is 40–60 bpm [29]. A minority (3.8%) of the 927 participants’ RHR were in the range of 101–115 bpm. Moreover, a significant difference in resting heart rate (*p* < 0.05) between gender groups was found. The resting heart rate for male (76.9 ± 12.5 bpm) was higher than that of female (74.1 ± 10.3 bpm). 

#### 3.2.2. Blood Glucose

Blood glucose is a major source of energy for most cells of the body, which is an indicator of diabetes self-management [30]. For the majority of healthy individuals, normal blood sugar level at fasting is less than 6.1 mmol/L and at random is less than 11.1 mmol/L. The glucose levels of 56 participants exceeded the thresholds. DMRT indicated a significant difference in glucose among age groups. The average glucose of the elderly (≥50 years) was 5.4 ± 1.6 mmol/L, which is higher than those between 35 and 50 years (4.9 ± 1.5 mmol/L) and those aged below 35 years (4.4 ± 0.7 mmol/L). 

#### 3.2.3. Liver Function

AST and ALT are reasonably sensitive indicators of liver damage or injury from different types of diseases or conditions. The normal range of AST is between 10 and 40 U/L, and ALT is between 7 and 56 U/L [31]. Mild elevations are generally considered to be two to three times higher than the normal range. While nine participants exceeded the AST level of 80 U/L, 13 participants exceeded the ALT level of 112 U/L. Moreover, a ratio of AST and ALT that exceeds 2 suggests alcoholic liver disease [32]. A total of 23 male participants had a ratio of AST and ALT more than 2. 12 of whom are problematic alcohol drinker. 

#### 3.2.4. Urea

The normal range of blood urea nitrogen (BUN) is between approximately 1.8 and 7.1 mmol/L [33]. A high BUN level indicates that the function of kidneys is not well [33]. The BUN of 151 participants is more than 7.1 mmol/L. Furthermore, the BUN for participants with more than seven daily sleeping hours (5.3 ± 1.1 mmol/L) was less than that of those with daily hours less than 7 h (5.9 ± 1.7 mmol/L). As a result, a significant difference in urea (*p* < 0.05) between sleeping habits was found.

#### 3.2.5. Cholesterol 

Maintaining the cholesterol levels within the ideal range is a great way to keep the heart healthy and lower the chances of having a heart disease or stroke [34]. The cholesterol level for 927 construction workers is 4.9 ± 0.8 mmol/L. According to the National Health Service (NHS) guidelines, it is recommended that the total cholesterol levels for healthy adults should be 5 mmol/L or less [35]. The cholesterol level of 399 participants exceeded this limit. DMRT indicated a significant difference in cholesterol level among age groups (Table 3). The average cholesterol of the elderly (≥50 years) was 5.3 ± 0.7 mmol/L, which is higher than those between 35 and 50 years (4.8 ± 0.9 mmol/L) and those aged below 35 years (4.5 ± 0.7 mmol/L).

#### 3.2.6. Uric Acid

The uric acid urine test in this research has two purposes: (i) to help diagnose the cause of recurrent kidney stones; and (ii) to monitor construction workers with gout at risk for stone formation. Normal uric acid levels are typically 140 µmol/L to 360 µmol/L for female and 200 µmol/L to 430 µmol/L for male [36]. High levels of uric acid are associated with gout, which is a form of arthritis that causes swelling of the joints, e.g., in the feet and big toes [37]. The uric acid level of 129 male participants exceeded 430 µmol/L, whereas the uric acid level of 22 female participants exceeded 360 µmol/L. DMRT indicated a significant difference in uric acid among age groups. The average uric acid of the elderly (≥50 years) was 381.7 ± 70.3 mmol/L, which is higher than those between 35 and 50 years (374.1 ± 78.2 mmol/L) and those aged below 35 years (342.5 ± 76.7 mmol/L). 

### 3.3. Musculoskeletal Pain 

#### 3.3.1. Pain Experience

A total of 901 participants completed the PEQ, in which 369 workers (312 male and 57 female) reported to have suffered pain/discomfort in the past three months. The majority (86.9%) of those workers who suffered pain were older than 45 years. The most frequently reported pain spots include the lower back (176 times, 37.6%), shoulder (93 times, 19.8%), knees (79 times, 16.8%), leg (67 times, 14.3%), and neck (53 times, 11.3%). Table 4 shows the severity of the pain (worst pain, slightest pain, and average pain) and present pain. The average pain of 369 workers who suffered pain symptoms is 3.72 ± 2.03. Meanwhile, a significant difference in average pain (*p* < 0.05) between alcohol drinking habits was found. Average pain for participants without alcohol intake (2.87 ± 2.25) was 23.0% lower than that for occasional alcohol drinking participants (3.53 ± 1.99) and was 42.1% lower than that for usual alcohol drinking participants (4.08 ± 1.83). Moreover, a significant difference in average pain (*p* < 0.05) between sleeping habits was found. The average pain for participants with more than seven daily sleeping hours (3.42 ± 1.91) was lower than that of those with less than 7 h of daily sleep (3.95 ± 1.87). Table 5 shows the effects of age, BMI, alcohol drinking habits, and working experience on musculoskeletal pain. Age and working experience explained 14.5% of the variability in the musculoskeletal pain. By adding BMI and alcohol drinking habit, the explained variability increased to 19.6%. Smoking habits were not statistically significant. 

#### 3.3.2. Pain Treatment

**Figure 3:**
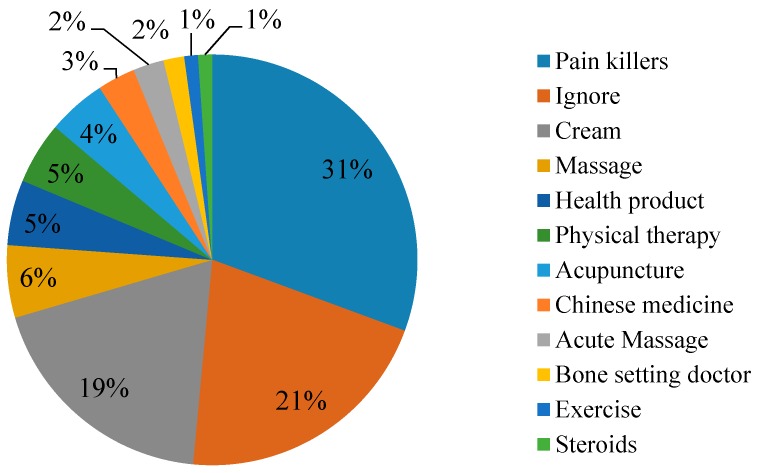
Shows the common methods for relieving pain symptoms used by the workers (sample size = 369).

Figure 3 shows the common methods for relieving pain symptoms used by the workers. The use of pain killers and relieving cream are the most commonly used method of construction workers in Hong Kong. Statistically, 133 workers adopted pain killers and 70 workers adopted cream to relieve pain/discomfort. However, 77 workers did not take any treatment for their pain. 

#### 3.3.3. Impacts of Pain on Daily Life 

The workers were asked to rate whether their pain symptoms have affected their mood, walking ability, work, relationships, sleep, and hobbies, on a seven-point Likert scale with anchors ranging from 1 (strongly disagree) to 7 (strongly agree). Table 6 indicates the ratings on the impacts of pain symptoms on workers’ daily living and its percentage in the different categories in the Likert scale. From the aspect of percentage in each category in the Likert scale, workers marked “agree” options (i.e., somewhat agree, agree, and strongly agree) in walking ability, work productivity, and sleep more frequently. From the aspect of the ratings, in general, the pain experience affects the work productivity and sleep of the workers, but not much of their relationship.

## 4. Discussion

In the present study, we examined the overall health condition and musculoskeletal pain of construction workers in Hong Kong. The current study found that approximately 40% of the participants’ BMI, cholesterol, and blood pressure exceeded threshold. Based in the estimates of Hong Kong Census and Statistics Department (2015), the prevalence of overweight and obesity in the construction industry was higher than in the general Hong Kong population with 36.1% vs. 20.8% and 6.5% vs. 2.0%, respectively [38]. Unfortunately, a high rate of overweight and obesity in the construction industry was considerably higher than in the general Dutch population with 64% vs. 51% and 15% vs. 10%, correspondingly. Moreover, the high rate of overweight and obesity in the construction industry is evident globally, with 75% in the Netherlands [39] and 63.7% in Germany [40]. 

More and more people in Hong Kong are living with hypertension and cardiovascular diseases. Surveys conducted by the Census and Statistics Department showed that the proportion of people with known hypertension increased from 9.3% in 2008 to 12.6% in 2014 [41]. According to a healthcare survey among 22,041 patients in Hong Kong between 2014 and 2015, almost 50% of them exceeded the healthy score for cholesterol [42]. The current findings, which show the percentage of the construction workforce with hypertension (39.8%) and elevated cholesterol concentrations (38.4%), are similar to those of the previous studies [43]. Gary et al. (2014) examined the health condition of steel workers in the United Kingdom and manifested that 40.7%, 15.8%, and 32.1% of the workers exceed the threshold of cholesterol, systolic blood pressure, and diastolic blood pressure respectively [43]. Phonrat et al. investigated the health status of road workers in Bangkok and showed that 15.8% male and 6.7% female exceed the threshold of systolic value, 38.6% male and 15.7% female exceed the threshold of diastolic value, and 57.9% male and 15.7% female exceed the threshold of cholesterol [44]. Excessively high level of cholesterol or persistent high blood pressure could increase the risk of heart attack, stroke, atherosclerosis, peripheral arterial disease, aortic aneurysms, kidney disease, and vascular dementia [45,46]. Previous studies identified the factors that can cause high cholesterol, including an unhealthy diet with too much saturated fat, smoking habit, and a family history of stroke or heart disease [47,48]. Several documents reported that people are at an increased risk of high blood pressure because of age (over 65 years), overweight, and unhealthy lifestyle (e.g., eating foods with too much salt, excessively drinking alcohol or coffee, insufficient intake of fruits and vegetables, lack of exercise, and lack of sleep) [47,49]. Construction workers, particularly elderly employees, should adopt a healthy lifestyle involving weight loss, decreased salt intake, physical exercise, and a healthy diet. 

Construction workers are found to be more susceptible of suffering from functional limitations and chronic diseases over the aging process [50]. The aging of the construction workforce poses a challenge in the Hong Kong construction industry. According to the statistics of Construction Workers Registration Board in July 2016, 42.5% of the registered workers are aged over 50 [51]. In contrast to employees in most other industries, construction workers usually start working at a very early age, e.g., 15 or 16 years old. As a result, when they are 50 years old, they have already been exposed to environmental demands and outdoor climate for 35 years. Therefore, the prolongation of working lives of construction workers should be seriously considered. 

This study reported that 41.0% of the participants (369/901) claimed to have experienced at least one MSD symptom in the past six months. On the contrary, previous studies showed that the prevalence of MSD symptom was higher than our findings. A study in Netherlands found that two-thirds of the 750 bricklayers suffered from musculoskeletal pain [52]. Similarly, 66.7% of construction workers in Malaysia reported such musculoskeletal complaint [53]. A survey conducted in United States among 200 cement and concrete workers indicated that 77% of the participants experienced at least one MSD in the past 12 months [17]. In Sweden, nearly 92% of the 1773 construction workers experienced musculoskeletal symptoms in the last year [54]. Moreover, workers in different trades and/or countries may have different degrees of susceptibility to MSD. Thus, an industry-specific study would better reflect the real situation. Nonetheless, the MSD symptom occurs in different body locations, such as the lower back, neck, shoulder, upper back, ankle, knee, upper arm, hip, forearm, and elbow. The current study shows that five body regions with the highest prevalence involved the lower back, shoulder, knees, leg, and neck, which is consistent with findings of earlier studies for MSD among construction workers. Low back pain was found to be the most frequent MSD among construction industry [17], particularly in concrete work, structural ironwork, roofers, and floorers [55,56]. On the other hand, neck disorders were prevalent among painters, insulators, and crane operators [56]. The major causes for work-related MSD symptoms may be attributed to the repetitive manual activities (e.g., transporting, lifting, or moving heavy materials or equipment) and longer duration of a stationary awkward posture [57]. Therefore, effective intervention strategies for eliminating/reducing the hazard of work-related musculoskeletal symptoms should be provided. One effective control measure is to implement the participatory ergonomics (PE) intervention. PE is defined as requiring the participation of those performing the work activities using a problem-solving approach to reduce the risk factors [57], which has been recognized as a useful strategy to decrease the burden of musculoskeletal pain, reduce psycho-social risk, and increase productivity [58]. Another control measure to reduce awkward postures and physical exertion is the adoption of an automatic apparatus. Vi et al. demonstrated the ergonomic/biomechanical benefits of utilizing the rebar-tying equipment, which is capable of reducing more than half of the peak low back loading [59]. Work safety climate is also important to the occupational health of construction workers. Those who perceived a less safe climate were at increased risk of experiencing musculoskeletal discomfort [60]. An investigation by Fung et al. found that construction workers do not realize the severity of musculoskeletal injuries [61]. Hence, both education and training on protecting workers’ from musculoskeletal injuries should be developed. 

This study pioneers the clinical examination and PEQ to assess the overall health status and musculoskeletal pain of construction workers in Hong Kong. A series of health parameters, such as BMI, blood pressure, glucose, cholesterol, uric acid, liver function, renal function, and musculoskeletal pain was evaluated. However, the limitation of the study is the sample size. This paper presents the preliminary findings of the Pilot Medical Examination Scheme (PMES) for construction workers between March 2014 and May 2015. Given that the scope of the PMES is to provide health examinations to 10,000 construction workers in the territory, further analysis on a larger sample size would be conducted. In this study, a self-reporting measure of musculoskeletal pain was employed, which underestimated or overestimated the prevalence of diagnosed MSDs. The relationship between personal, lifestyle, and work-related factors and musculoskeletal pain was thereby explored. Apart from age, BMI, alcohol drinking habit, and working experience, several factors such as physical load and work-related psychosocial load might influence musculoskeletal pain. Thus, further analysis by integrating other work-related factors is envisaged. 

Construction health and safety is an important issue worldwide. A large number of construction workers drop out of the sector early because of injuries, ill health or permanent disabilities. Although this study applies specifically to the Hong Kong construction industry, the same research methodology could be extended to other regions/countries and to other sectors to detect the occupational health condition of the workforce and develop appropriate intervention strategies.

## 5. Conclusions

The construction industry is recognized as one of the most dangerous industries. Numerous efforts have been devoted to reduce safety hazards; however, less attention has been given to occupational health issues. In Hong Kong, a PMES was launched to help identify the health profile of construction workers. Based on the medical examinations and questionnaire survey, it is found that 31.1% and 6.5% of the participants were moderately overweight and obese, respectively; 39.8% and 38.4% of the participants exceeded threshold for cholesterol level and blood pressure, correspondingly; and 39.2% of the participants suffered musculoskeletal pain. Thus, a collaboration among workers, client owners, contractors, and government to initiate occupational health trainings and educational systems for the improvement of the health status of construction workers could be useful. Such programs involve healthy lifestyle, occupational health work practices, ergonomic principles, and personal protection in the training curriculum. Specifically, the government and industry could launch an occupational health scheme in terms of maintaining healthy lifestyle and promoting safety culture in the construction industry. Clients and contractors could implement control measures, such as redesigning the work station to reduce the work load of muscular system, providing personal protective equipment (e.g., back support belt), introducing mechanical aids, and allowing regular breaks to avoid a stationary work status. Frontline workers should also pay attention to and comply with the guidelines and practice notes as well as participate in occupational health activities. 

The scope of PMES is to provide basic health examinations for 10,000 workers in the construction industry of Hong Kong, and, to date, we have examined 942 construction workers. Based on the data obtained from the 942 workers, we have undertaken some preliminary analyses and reported the results. Future studies need to be done in a larger sample size. Moreover, similar studies should be carried out on a regular basis to monitor the health profile of construction workers longitudinally.

## Figures and Tables

**Table 1:** Demographic details of participants of the PMES (sample size = 942).

Characteristic	*N*	Min	Max	Mean ± Standard Deviation (SD)	Percentage (%)
Age (years)	942	17	70	45.1 ± 12.1	-
Male	818	17	70	44.3 ± 12.4	-
Female	124	28	66	50.5 ± 12.1	-
Weight (kg)	932	42	115	68.5 ± 11.6	-
Height (cm)	932	145	191	168.6 ± 8.0	-
Body Mass Index (BMI) (kg/m^2^)	932	13.3	36.8	24.3 ± 3.7	-
Sleeping (hs)	938	4	11	6.9 ± 1.2	-
Smoking Habits	-	-	-	-	-
Non-Smoker (%)	477	-	-	-	50.9
Occasional Smoker (%)	101	-	-	-	10.8
Daily Smoker (%)	360	-	-	-	38.4
Alcohol Drinking Habit	-	-	-	-	-
Non-Alcohol Drinker (%)	380	-	-	-	40.4
Occasional Alcohol Drinker (%)	494	-	-	-	52.7
Problematic Alcohol Drinker (%)	64	-	-	-	6.8
Ethnicity	-	-	-	-	-
Hong Kong (%)	839	-	-	-	89.4
Mainland Chinese (%)	87	-	-	-	9.3
Pakistani (%)	6	-	-	-	0.6
Nepalese (%)	6	-	-	-	0.6
Working Experience in Construction (years)	939	0.2	53	12.9 ± 10.9	-

**Table 2:** Health parameters of the Hong Kong construction workers (sample size = 927).

Parameter	Mean ± SD
DBP (mmHg)	77.7 ± 14.5
SBP (mmHg)	135.3 ± 17.2
Resting Heart Rate (bpm)	76.7 ± 12.1
Glucose-Fasting (mmol/L)	4.9 ± 1.4
Glucose-Random (mmol/L)	5.7 ± 2.2
AST (U/L)	30.2 ± 16.4
ALT (U/L)	33.3 ± 20.9
Urea (nmol/L)	5.7 ± 1.5
Cholesterol	4.9 ± 0.8
Uric Acid	360.3 ± 81.7

DBP = Diastolic Blood Pressure; SBP = systolic blood pressure; AST = aspartate aminotransferase; ALT = alanine aminotransferase.

**Table 3:** Comparison of means of health parameters in age groups ^a^.

Health Parameters	Mean	SD	*F*	Significance
Glucose (Fasting) (mmol/L) ^b^	-	-	-	-
<35 years	4.4	0.7	8.66	0.002
35–50 years	4.9	1.5	-	-
≥50 years	5.4	1.6	-	-
Cholesterol (mmol/L) ^b^	-	-	-	-
<35 years	4.5	0.7	46.76	0.000
35–50 years	4.8	0.9	-	-
≥50 years	5.3	0.7	-	-
Uric Acid (mmol/L) ^c^	-	-	-	-
<35 years	342.5	76.7	19.43	0.001
35–50 years	374.1	78.2	-	-
≥50 years	381.7	70.3	-	-

^a^ One-way ANOVA and Duncan’s multiple range test (DMRT) by age groups (≤35 years, *n* = 221; 35–50 years, *n* = 435; ≥50 years, *n* = 281); ^b^ DMRT: participants younger than 35 years, participants between 35 and 50 years, participants older than 50 years are significantly different; ^c^ DMRT: participants between 35 and 50 years, participants older than 50 years are significantly different.

**Table 4:** Pain severity ^a^ of the Hong Kong construction workers (Mean ± SD).

Pain Severity	Male	Female	Pooled
Sample size	312	57	369
Most Intense Pain over the Past 24 h	5.5 ± 2.6	4.9 ± 1.8	5.2 ± 2.5
Slightest Pain over the Past 24 h	2.1 ± 1.8	1.9 ± 2.0	2.0 ± 1.9
Average Pain over the Past 24 h	3.7 ± 2.2	4.2 ± 1.9	3.9 ± 2.0
Present Pain	2.5 ± 2.3	2.9 ± 2.1	2.6 ± 2.5

^a^ 0 is no pain, 1 is very mild, 2 is discomforting, 3 is tolerable, 4 is distressing, 5 is very distressing, 6 is intense, 7 is very intense, 8 is utterly horrible, 9 is excruciating unbearable, 10 is unimaginable unspeakable.

**Table 5:** Multivariate analysis of the relationship between musculoskeletal pain and age, BMI, alcohol drinking habits, working experience among construction workers in Hong Kong.

Characteristic	Estimate	Standard Error	Significance
Age (years)	−0.14	0.003	0.000
Working Experiences (years)	−1.17	0.054	0.023
BMI	-	-	-
Underweight	Reference	0.000	0.000
Normal	0.02	0.036	0.024
Overweight	−0.48	0.067	0.009
Obese	−0.94	0.065	0.003
Alcohol Drinking Habit	-	-	-
Non-Drinker	Reference	0.000	0.000
Occasional Alcohol Drinker	−0.13	0.082	0.034
Problematic Alcohol Drinker	−0.65	0.099	0.022

**Table 6:** Ratings on impacts of pain on workers’ daily living and its percentage in the different categories in the Likert scale (sample size = 269).

Item	1	2	3	4	5	6	7	Mean ± SD
	Strongly Disagree	Disagree	Somewhat Disagree	Neither Agree Nor Disagree	Somewhat Agree	Agree	Strongly Agree	
Mood	1	50	77	73	51	14	2	3.6 ± 1.2
Walking Ability	3	12	35	72	77	67	2	4.5 ± 1.2
Work Productivity	4	10	39	36	76	65	38	4.9 ± 1.5
Relationship between Family, Friends, Partners	41	80	57	46	32	9	3	2.9 ± 1.4
Sleep	7	26	23	34	75	78	25	4.8 ± 1.5
Hobbies	43	63	56	13	43	28	22	3.4 ± 1.9
